# Father and Son Jointly Announced the Discovery of Röntgen Rays in Two Consecutive Press Articles – Revisited

**DOI:** 10.5334/jbsr.2919

**Published:** 2023-01-04

**Authors:** Jean-François Monville, Robert F. Dondelinger

**Affiliations:** 1 St. Nikolaus Hospital Eupen, BE; 2 University of Liège, BE

**Keywords:** Röntgen, 1896, radiology history, x-rays, discovery

## Abstract

It is generally omitted that the press article announcing the discovery of Röntgen rays on the 5^th^ of January 1896 was followed by a second article published two days later in the same Viennese newspaper *Die Presse* under the same heading. While the initial article was composed hastily by the editor in a journalistic style and contained no information on the nature of the new rays, the second publication was partly composed by the son of the editor, a physicist, who informed on some of the basic physical properties of the Röntgen rays.

It is widely known that the announcement of the discovery of X-rays was published by the general press for the first time in the morning edition of the Viennese daily journal *Die Presse* on Sunday January 5,1896 [1]. January 6 was a legal and religious holiday in Austria without distribution of newspapers. The next issue of *Die Presse* became available on Tuesday morning January 7. It is generally neglected that this issue contained a continuation of the initial paper under the identical heading ‘A Sensational Discovery’ [2]. The editorial lay-out of both papers was almost the same. It started with the last column on the front page to catch the eye of the reader and continued overleaf. The second article was shorter by one third. Both press communications were not signed, according to the editorial rule of the journal. The publication of Tuesday’s edition mentioned “Vienna, January 6^th^’’ underneath the heading in line with journalistic practice. The reason for the addition was to notify the reader that the published material originated from a source located in Vienna and was collected after the preceding issue had gone to print. (Figure 1 a, b) Soon, the anteriority of the first article overshadowed the second, despite the latter was more informative.

**Figure 1:**
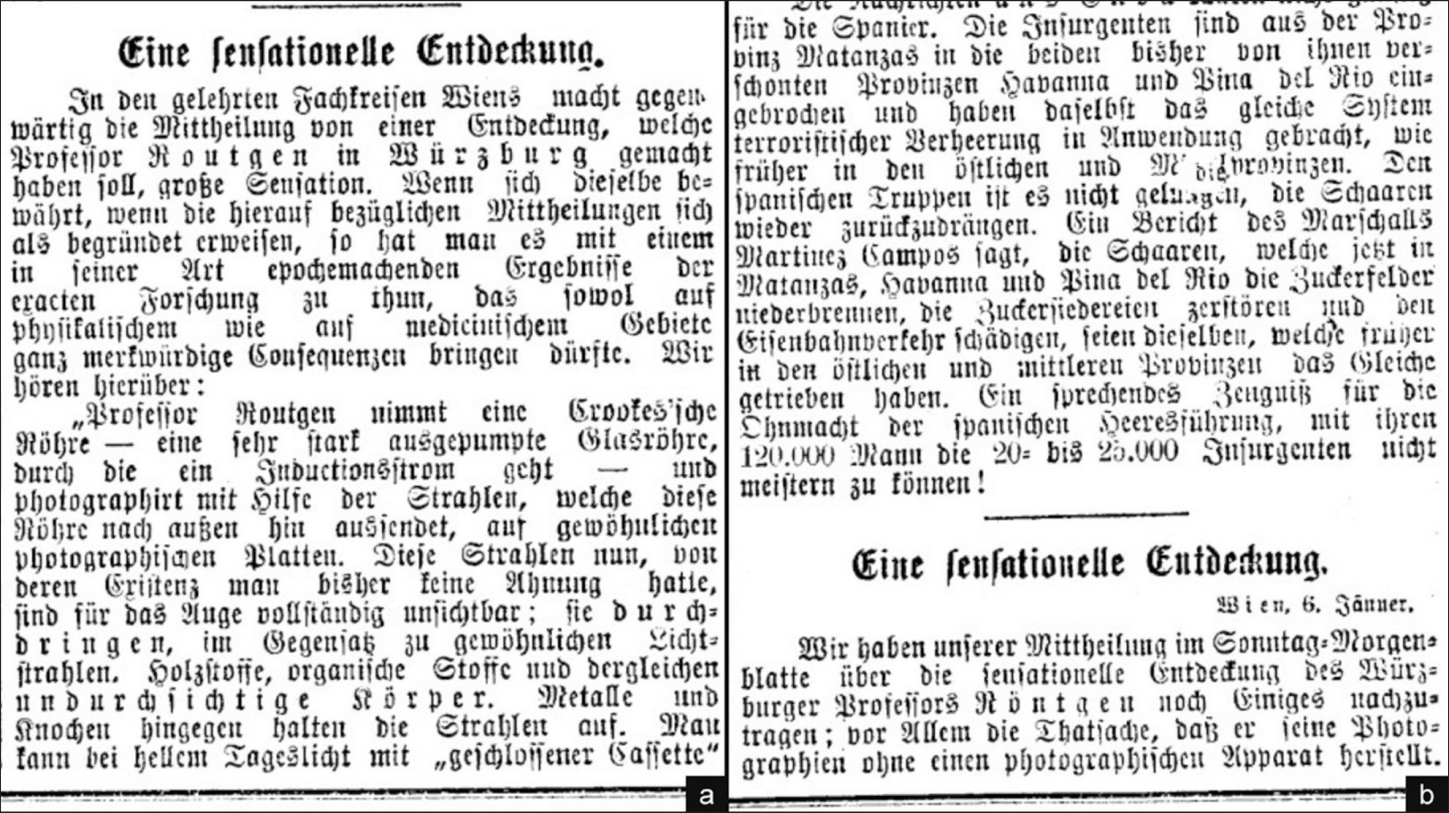
Die *Presse*, Vienna. Front page headlines from January 5 **(a)** and 7 **(b)**, 1896 [1,2].

Zacharias Konrad Lecher (1829–1905), (Figure 2) a recognized pressman of Vienna, was the editor (*Herausgeber*) of *Die Presse* from 11.04.1871 to 31.10.1896, when the journal ceased publication. Ernst Lecher (1856–1926) (Figure 3), the elder of two sons of Zacharias L., was an ordinary professor of experimental physics at the German University of Prague. He sojourned in Vienna around the turn of the year 1895 for the Christmas vacation. On the Saturday evening January 4, 1896, Ernst L. attended one of the regular social gatherings held by Franz-Serafin Exner Jn. (1849–1926) (Figure 4) at his home at Währingerstrasse, 29 [3]. Exner was an ordinary professor at the University of Vienna and head of the chemical physical institute since 1891 in Türkenstrasse, 3. He became acquainted with Röntgen (1845–1923) during a common apprenticeship in Zurich around 1869/1870 and thereafter in Strasbourg [4]. Exner exhibited to his circle of guests an off-print of Röntgen’s recently published prime article entitled ‘On a new kind of rays. A preliminary communication’ [5]. He showed around nine accompanying photographic prints that Röntgen had obtained by the new rays [2]. The mail that had been sent out by Röntgen from Würzburg no earlier than Wednesday January 1, 1896, to a selection of prominent scientists in Europe, had reached Exner in Vienna before the Saturday evening debate took place. This was not unusual postal speed at the end of the nineteenth century. Two physicists, Stokes (1819–1903) in Cambridge and Warburg (1846–1931) in Berlin confirmed in a letter to having received the same mail on January 3 [6]. Warburg took advantage of the celebration of the fiftieth anniversary of the Berlin Society of Physics to exhibit Röntgen’s photographs to the members of the Society and their guests [7]. However, the sensational documentation went more or less unnoticed by the unprepared minds of visitors. In Vienna, later that same day, Ernst L. pointed out to his father the importance of Röntgen’s discovery. Relying on veracity of his son’s revelation, Zacharias L. decided on immediate publication of the spectacular news in the next morning’s issue. In his capacity of editor, he adapted the lay-out of the front page to accommodate a last-minute article, written up during the night. For the sake of urgent typesetting, Zacharias L. probably completed his paper at the pressroom and printery of ‘Die Presse’, located in Berggasse, 31, a relatively short distance from Exner’s house. This news story was obviously set up in a hurry. The name of Röntgen was misspelled ‘Routgen’. It seems likely that Exner had entrusted his personal off-print and Röntgen’s photographs to Ernst L. to serve as reference material to his father. Indeed, Exner complained in a letter to Röntgen on January 11, that the photographs were always in the hands of other people and not his own [6]. The resulting press article was made of two parts. It is out of the question that an employee of *Die Presse* had written it up instead of the editor. The uniform journalistic style strongly suggests that Zacharias L. was the sole author of the entire paper. In the first part, the father reported rather briefly with quotation marks the testimony made by the son. The sources, namely his son and Exner were not revealed. In one sentence, Röntgen rays are called ‘light rays’ (*Lichtstrahlen*). It is true that Röntgen questioned dubitative similarities of light rays or ultraviolet rays and X-rays. Zacharias L. described shortly and in layman’s terms, Röntgen’s experiment with a Crookes’ tube producing the photographic effect. The author put most emphasis on the fact that the photographs were taken in daylight conditions without direct exposure of the photosensitive film, which remained protected inside a closed wooden box. Röntgen rays pierced wood but did not penetrate a set of measuring weights (Figure 5) contained in a box and other various metal objects. He finished the chapter with the description of ‘the most surprising photograph of a human hand’. The picture showed the bones of the hand and rings seem to float freely around the finger. The soft tissues were not ‘visible’. Correct identification of two radio-opaque rings on the fourth finger was self-evident. Comparison with a portrait photograph suggests that the rings shown on the radiograph were worn by Röntgen’s wife, Anna Bertha (1839–1919) [8]. Of Röntgen’s mailings, only two complete and identical sets of nine photographs have been preserved. Those which Röntgen had sent to the physicists Schuster (1851–1934) in Manchester [9] and Lorentz (1853–1928) in Leiden [10]. The set of prints received by Exner did not come to us, so far. Only two of the most striking photographs were explicitly quoted in Zacharias’ L. article: the set of measuring weights and the human hand. The first photograph was not included in the two historical sets, but its existence was mentioned in Röntgen’s manuscript. Knowing that Exner had received nine photographs and assuming that his set was identical to that of Schuster and Lorentz, the question arises, where did this photograph come from? Did Röntgen have contact with Exner on a previous date, revealing his discovery on a confidential basis, before the submission of his preliminary communication? There is no proven evidence of such a conjecture which has been raised by some, but seems unlikely [11]. The photograph of measuring weights kept by the German Röntgen museum is dated 25 Dec 1895 [6]. It is more tenable that the pieces of the photographic sets sent out varied a little for irrelevant practical reasons or simply due to the availability of prints. The photographs exhibited by Warburg on January 4, also included a set of measuring weights [12]. When Röntgen provided the physicist Kohlrausch (1855–1936) in Hannover with additional prints on January 15, 1896, he explained that he was able to send photographs at a first shot only to Warburg and to Exner, and he complained that he had had to do it all on his own, as his assistant had left for Christmas vacation [6]. We must therefore admit that Röntgen’s photographic sets were simply not all identical.

**Figure 2:**
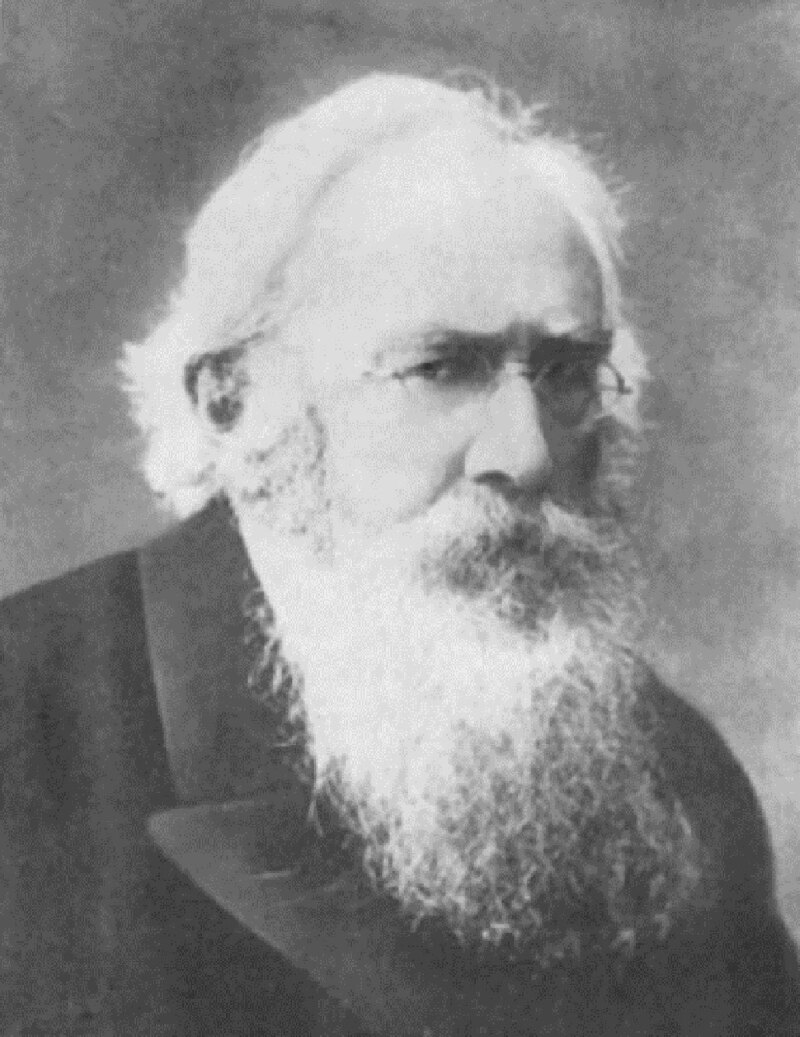
Zacharias Konrad Lecher (1829–1905) [13].

**Figure 3:**
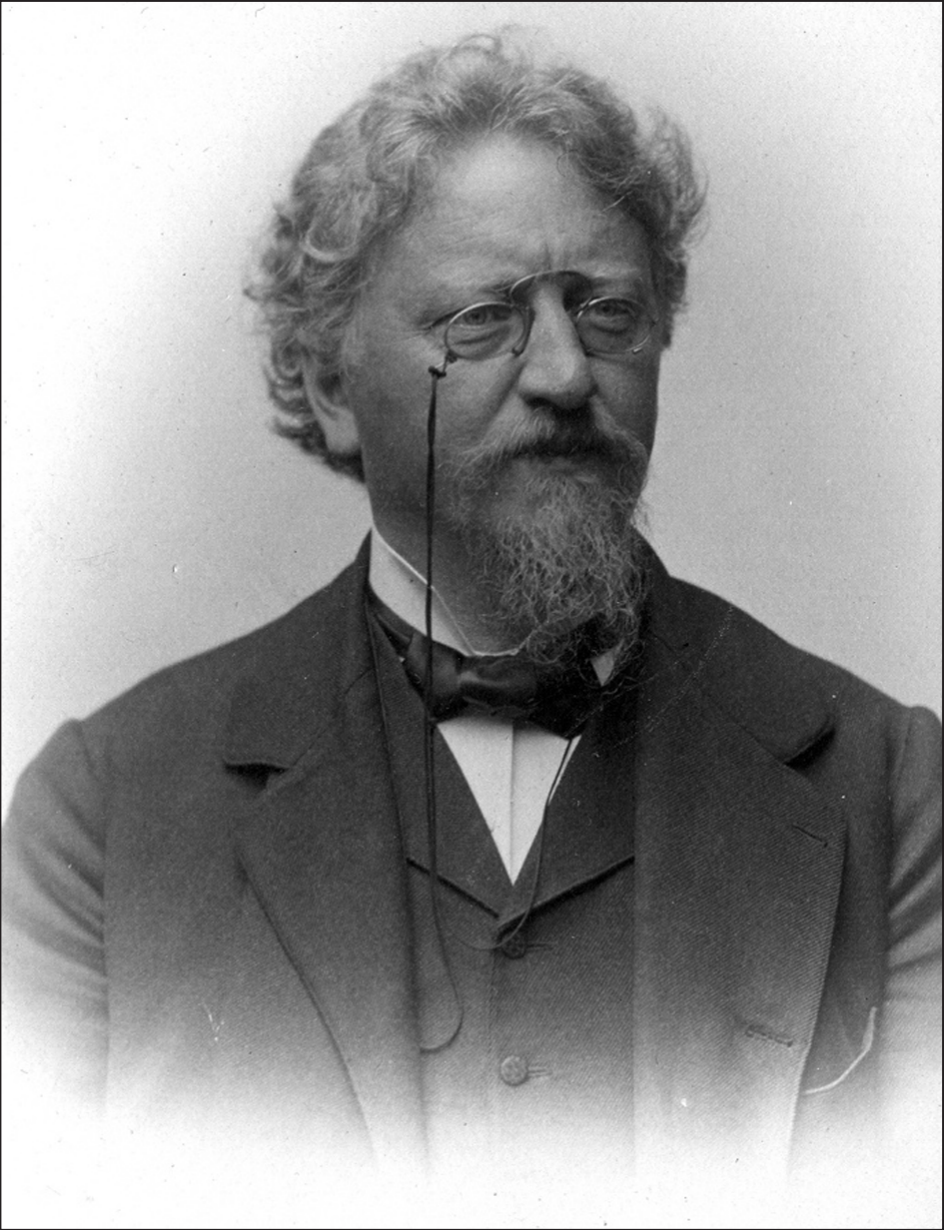
Ernst Lecher (1856–1826) [15].

**Figure 4:**
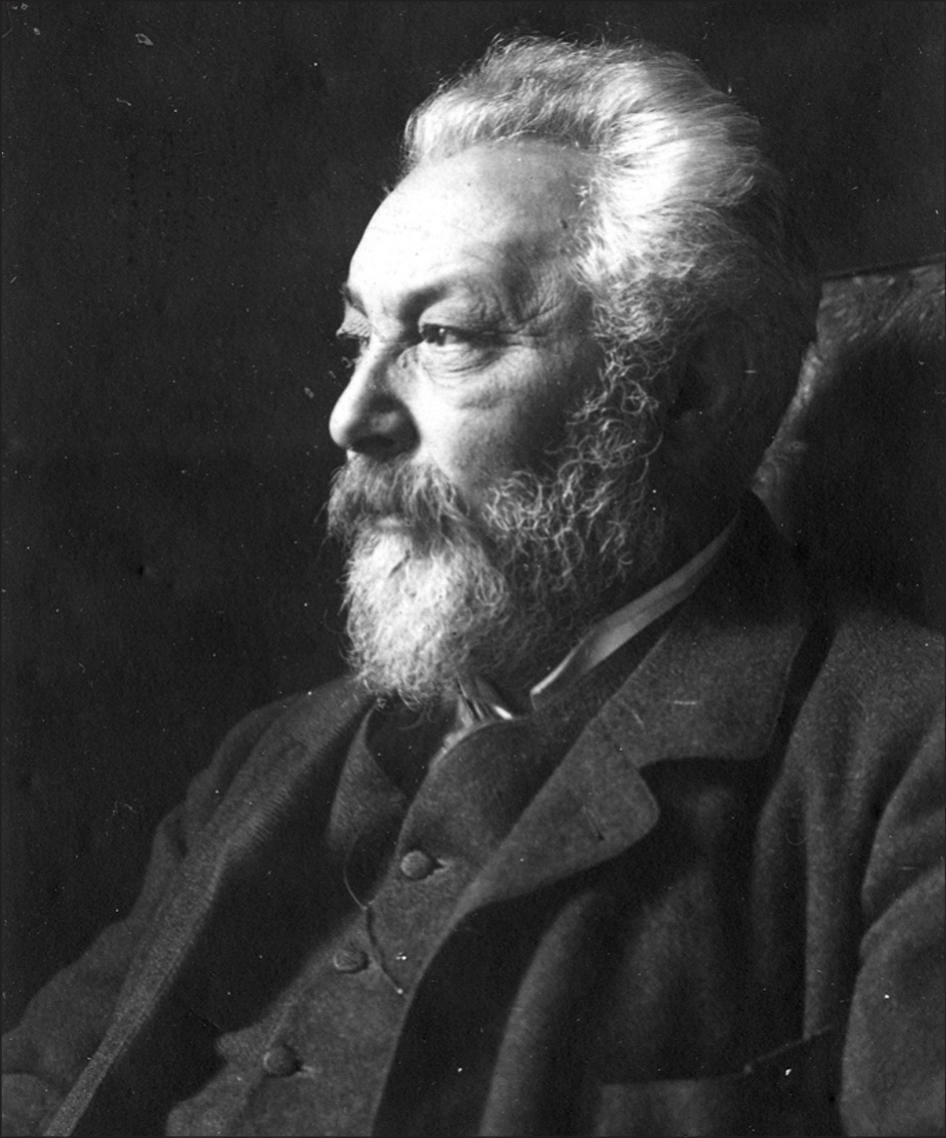
Franz-Serafin Exner (1848–1926) (University of Vienna. Picture archive).

**Figure 5:**
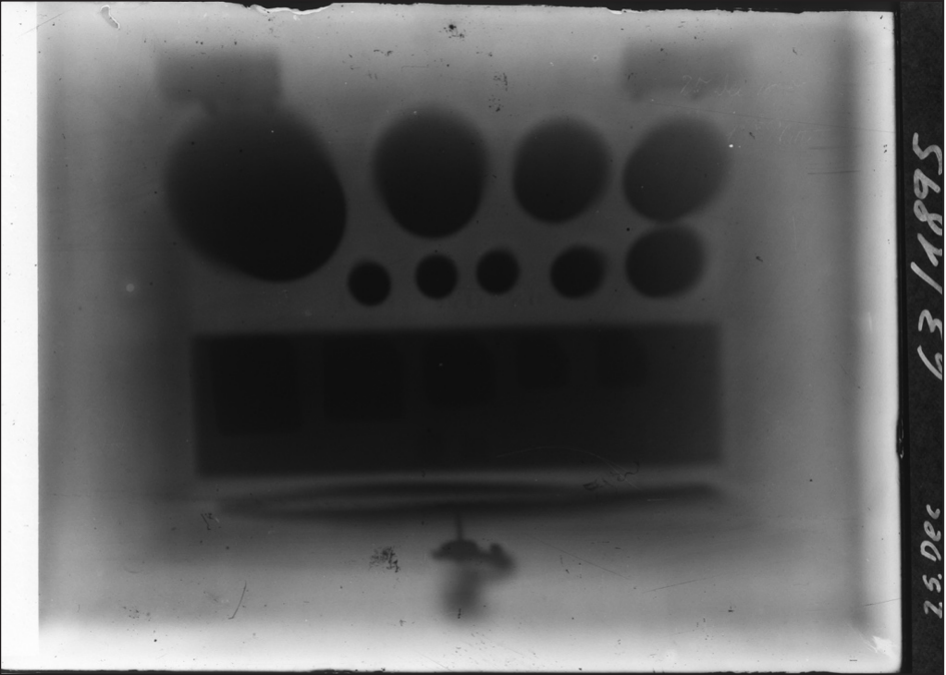
Set of measuring weights. Dated 25 Dec – 63/1895 [6].

In the predominant second part of the article, Zacharias L. commented extensively in a rather enthusiastic journalistic style on future applications and development of the new rays. He listed physicists, photographers, biologists, physicians and in particular surgeons as professional groups with a potential interest in the application of the promising discovery. Biologists were included in the listing, as they were usually targeted together with physicians by textbooks of medical physics of the time. Zacharias L. was the son of a medical practitioner, Michael Lecher (1802–1853) and in his youth he was in contact with veterinary medicine and biological sciences in Munich for a short time [13]. Memories from his father’s practice, personal background knowledge, a dose of common sense and a summary of the evening discussion reported by the son, were enough to enable Zacharias L. to foresee the medical use of Röntgen rays. The journalist emphasized the importance of imaging bone structures for diagnosis of complex fractures, for diagnosis and therapy of non traumatic bone diseases and localisation of foreign bodies. It is not excluded that the elder brother of Exner, Sigmund Exner (1846–1926), ordinary professor of physiology in 1891, had been present at the memorable Saturday evening party and had fueled the discussion on the future medical employment of Röntgen rays. In a last chapter, the power of imagination of Zacharias L. was at its best. He formulated in unequivocal wording the astonishing prophecy of radiographic tomography, which in fact, came into existence only some thirty years later [14]. At the end, Zacharias L. concluded in his bloomy style, comparing the significance of Röntgen’s innovation with: ‘Whoever had predicted at the beginning of this century, that the generation of our grandchildren would be able to shoot clear pictures of a flying bullet was at risk to be under suspicion moving towards the madhouse’. The father hinted here at the scientific physical work of Mach (1838–1916), whom Ernst L. had recently succeeded in his academic position in Prague [15]. An indirect allusion to the academic promotion of his son can be assumed.

The next article, published on January 7 [2], contrasted in content and style with the foregoing hasty one. It is patent that the authors, Ernst L in particular, were not satisfied with their half-baked text, which had entirely neglected the physical aspects of Röntgen’s discovery, highlighting the sensational photographs, without saying anything about the nature of the rays. It is obvious that it must have been Ernst L. who insisted on his father reviewing their copy and adding a description of the most prominent physical properties of Röntgen rays, giving real value to the vague information that had been sent ahead. The tone of the first paragraph is scientifically sober, style is deprived of showmanship, journalistic paraphrasing or speculation. The reader learned the following:

Above all, Röntgen obtained his photographs without a photographic device, because the rays cannot be concentrated by a lens, as they are not deflected. They fall directly on the object and impress the photographic paper which is enshrined in a box placed behind or under the object. The protective wooden cover of the box can remain closed. Röntgen rays are invisible to the eye, they do not produce heat and are not influenced by a magnetic field. They do not move in wave form, but along straight lines. Such a distinctive property of aetheric movement has been hypothesized by physicists, but so far has never been proven. The observation of rays running out along a straight line is much more significant than their photographic effect, which appears as a secondary attribute.

This mini-lecture on basic physics, undoubtedly came from the pen of Ernst L. with the intention to ground the sensational announcement made by his father two days before on scientific evidence and place the X-rays in the perspective of a physicist. The photographic effect of the new rays was of secondary importance to physicists, as other deeply investigated rays were able to do the same.

The two last paragraphs seem to be written again by Zacharias L. in his personal literary style, but nourished by facts reported by his son. Zacharias L. asserted that ‘we’, he meant his son and other physicists ‘were able to convince ourselves that Röntgen’s first communication corresponds absolutely to the truth.’ He testified that nine photographs were sent by Röntgen to a local prominent physicist, without naming Exner. Zacharias L. insisted on the fact that Röntgen’s photographic material proved real, resisting the most intense scrutiny by experts. The best proof of two writers being at work here is that the photographic process was described once more. Concerning the radiograph of the hand, it is interesting to notice the article saying that Röntgen put the hand on the film during exposure. However, when Röntgen was asked during an interview published in April 1896, to reproduce his first experiment, Röntgen placed the hand on the table and the plate on his hand, while the tube fired from under the table [16].

The last paragraph contains somewhat puzzling information at a first glance. Zacharias L. reported on the circumstances of the discovery itself, telling the reader that Röntgen serendipitously noticed the fluorescent effect of the new rays. His Crookes’ tube was wrapped with fabric, preventing light from interfering. When the tube was put under tension, Röntgen observed lines which appeared on a prepared reception paper. Those lines were not created by the electric current and the receptive paper was disposed at a too great distance to be reached by cathode rays. How did Ernst L. get this information on January 5 or 6? First, Röntgen described in his manuscript that the tube was wrapped with black cardboard. He might, however, have used fabric initially, which was at hand in the laboratory. Second, emergent lines were not described in Röntgen’s manuscript. Valid explanations for both incongruities exist. Concerning the use of fabric, Röntgen certainly had included a personal letter to his youthful friend Exner, in which he may have released detailed information on his first experimental observation and mentioned fabric, or the authors of the paper simply confused cardboard with fabric. Concerning the lines detected on the fluorescent support, either Röntgen reported on the conditions of his first observation in the letter or Exner and his co-workers had already tried to produce X-rays between January 4 and 6, observing linear shadows created by some object or hand skeleton interposed between the radiating tube and a fluorescent surface. Father and son may simply have imagined what was the signal observed on the fluorescent screen. All explanations are plausible. Indeed, Zacharias L. alluded to such trials as he closed the article finalized on January 6 by stating: ‘It seems so far that the making of Röntgen’s photographs was not successful [by Viennese physicists], because the required equipment is not powerful enough’. This proves that experimentation started in Vienna directly following the receipt of Röntgen’s material. Ernst L. had either witnessed firsthand this progress or he had heard about it during the previous two days. Even if the Crookes’ tubes available in Exner’s laboratory or elsewhere in Vienna were too feeble to produce discriminative photographic shadows, they might have been strong enough to excite to some degree fluorescent material placed in the vicinity of the radiant source. After contact with other researchers on the Sunday and Monday, Ernst L. was able to come back with the latest news, which found again their place starting on the front page of his father’s newspaper. On January 11, Exner confessed to Röntgen in a letter, that he still failed reproducing the experiment although he guessed, it must be quite simple [6]. During the following months, Ernst L. did not show interest in further investigation of the Röntgen rays.
